# Machine learning-aided search for ligands of P2Y_6_ and other P2Y receptors

**DOI:** 10.1007/s11302-024-10003-4

**Published:** 2024-03-25

**Authors:** Ana C. Puhl, Sarah A. Lewicki, Zhan-Guo Gao, Asmita Pramanik, Vadim Makarov, Sean Ekins, Kenneth A. Jacobson

**Affiliations:** 1 Collaborations Pharmaceuticals, Inc, 840 Main Campus Drive, Lab 3510, Raleigh, NC 27606 USA; 2 Molecular Recognition Section, Laboratory of Bioorganic Chemistry, National Institute of Diabetes and Digestive and Kidney Diseases, National Institutes of Health, Bethesda, MD 20892 USA; 3 Research Center of Biotechnology RAS, Leninsky Prospekt 33-2, 119071 Moscow, Russian Federation

**Keywords:** Machine learning, Drug discovery, Purinergic, Receptor, Uracil nucleotides

## Abstract

The P2Y_6_ receptor, activated by uridine diphosphate (UDP), is a target for antagonists in inflammatory, neurodegenerative, and metabolic disorders, yet few potent and selective antagonists are known to date. This prompted us to use machine learning as a novel approach to aid ligand discovery, with pharmacological evaluation at three P2YR subtypes: initially P2Y_6_ and subsequently P2Y_1_ and P2Y_14_. Relying on extensive published data for P2Y_6_R agonists, we generated and validated an array of classification machine learning model using the algorithms deep learning (DL), adaboost classifier (ada), Bernoulli NB (bnb), *k*-nearest neighbors (kNN) classifier, logistic regression (lreg), random forest classifier (rf), support vector classification (SVC), and XGBoost (XGB) classifier models, and the common consensus was applied to molecular selection of 21 diverse structures. Compounds were screened using human P2Y_6_R-induced functional calcium transients in transfected 1321N1 astrocytoma cells and fluorescent binding inhibition at closely related hP2Y_14_R expressed in CHO cells. The hit compound ABBV-744, an experimental anticancer drug with a 6-methyl-7-oxo-6,7-dihydro-1*H*-pyrrolo[2,3-*c*]pyridine scaffold, had multifaceted interactions with the P2YR family: hP2Y_6_R inhibition in a non-surmountable fashion, suggesting that noncompetitive antagonism, and hP2Y_1_R enhancement, but not hP2Y_14_R binding inhibition. Other machine learning-selected compounds were either weak (experimental anti-asthmatic drug AZD5423 with a phenyl-1*H*-indazole scaffold) or inactive in inhibiting the hP2Y_6_R. Experimental drugs TAK-593 and GSK1070916 (100 µM) inhibited P2Y_14_R fluorescent binding by 50% and 38%, respectively, and all other compounds by < 20%. Thus, machine learning has led the way toward revealing previously unknown modulators of several P2YR subtypes that have varied effects.

## Introduction

G protein-coupled receptors (GPCRs) are important pharmaceutical targets comprising the single largest structural family of gene products in the human genome and are characterized by seven transmembrane helices (TMs) [1]. Various computational approaches have been applied to the discovery of new GPCR ligands [2–5]. Structure-based approaches have sampled chemical space broadly to reveal new chemotypes as agonists or antagonists of various GPCRs, which may then be optimized structurally. Computational approaches for GPCR ligand discovery may be generally more efficient than high throughput screening of assembled chemical libraries. Another productive approach is to use GPCR structure-based or homology models and docking/molecular dynamics to guide the modification of known ligands by rational design [6]. Recently, machine learning (ML) has become a promising tool in medicinal chemistry for systematic drug discovery, in general, and with respect to GPCRs specifically [5]. ML techniques harness the power of algorithms to analyze vast structure activity relationship datasets, recognize patterns, and make predictions based on learned molecular features. ML has been applied to rapidly screen chemical databases, predict molecular interactions, and identify potential ligands [7–9] and can be used literally for end-to-end across drug discovery [7]. But the full potential of ML for GPCR ligand discovery has yet to be demonstrated [10].

**Fig. 1:**
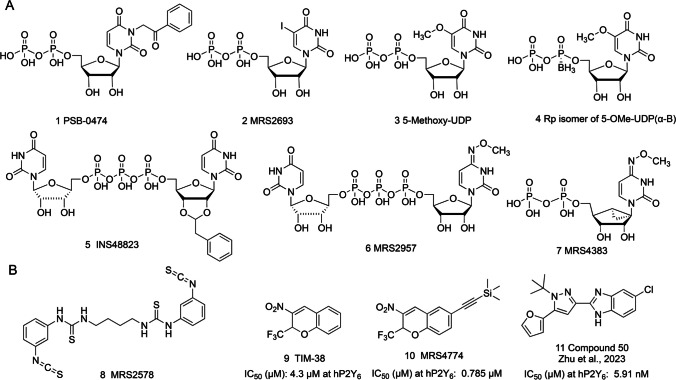
Known ligands of the P2Y_6_R with approximate affinity values. **A** Agonists. **B** Antagonists. See references [11, 22–26, 28–30] for details

In this study, we have focused our efforts on the modulators of purinergic signaling, an extensive signaling system relevant to many pathological conditions and the focus of drug discovery efforts. There are 19 cell-surface receptors in total in the signaling “purinome,” including 12 GPCRs (8 P2Y and 4 adenosine receptors). Among the P2Y purinergic receptors that have attracted interest for future therapeutics [11], the G_q_ protein-coupled P2Y_6_ receptor (P2Y_6_R) has emerged as an intriguing target due to its role in diverse physiological processes, including cell proliferation, inflammation, cerebroprotection, and immune responses [12, 13]. Activation of the P2Y_6_R has been implicated in various pathological conditions, including Alzheimer’s disease, Parkinson’s disease, epilepsy, pulmonary inflammation and fibrosis, diabetes, cardiovascular disease, and chronic neuropathic pain [12, 14–21]. Thus, it is an attractive target for the development of antagonists as novel pharmaceutical agents. However, there is no P2Y_6_R structure available, and there are currently few selective P2Y_6_R antagonists (Fig. 1). In contrast, the structure activity relationship (SAR) of P2Y_6_R agonists, including those with high affinity, has been reported [22–25]. The most frequently used P2Y_6_R antagonist in pharmacological studies is MRS2578, which is an irreversibly binding diisothiocyanate derivative [26, 27]. Chromene derivatives have also been explored as P2Y_6_R antagonists but are of only moderate affinity [28, 29]. Recently, Zhu et al. [30] reported apparently competitive P2Y_6_R antagonists in the class of 2-(1-(*tert*-butyl)-5-(furan-2-yl)-1*H*-pyrazol-3-yl)-1*H*-benzo[d]imidazole derivatives. Representative X-ray crystallographic [31] and cryo-electron microscopic (cryo-EM) [32] structures of the P2Y_1_R, a member of the same G_q_-coupled P2YR subfamily (P2Y_1_-like), are available and have been used as a template for modeling other related P2YRs [30, 33, 34]. In this study, we evaluated the interaction of the ML-identified compounds at three P2YR subtypes: the uracil nucleotide-preferring P2Y_6_R and P2Y_14_R and the adenine nucleotide-preferring P2Y_1_R. All three subtypes recognize nucleoside 5′-diphosphates as endogenous ligands and are related to inflammatory pathways [11]. Thus, antagonists could have translational potential.

## Materials and methods

### Data curation and machine learning modeling

Public data available on P2Y_6_R agonists in ChEMBL [35] was found at ChEMBL4714 which was curated and used to build ML models. Collaborations Pharmaceuticals’ proprietary software “E-Clean” was used to “clean” and average activities for datasets prior to model building in Assay Central (AC) [36]. “E-Clean” handles duplicate compounds by either averaging, removing, or keeping duplicates based on InChIKey. For these data, duplicate molecules with continuous activity data were first converted to -logM and then were averaged. “E-Clean” logs the SMILES strings of the duplicate compounds along with their activities and indices for inspection by the user. If needed, compounds were also subjected to charge neutralization, salt removal, and standardization via custom software using open-source RDKit functions. The standardization within AC was done as follows: A simple standardization workflow consisting of the following steps and using the Indigo Toolkit was applied: read molecule from the string representation (e.g., SMILES or MOL), generate InChI and InChIKey, use InChIKey to find and remove duplicates, dearomatize, remove enhanced stereo, remove unknown stereo, standardize and reposition, if necessary, stereo bonds (e.g., wedged bonds), standardize or flag erroneous charges, flag erroneous valences, remove isotopes, remove dative and hydrogen bonds, remove smaller component if multicomponent chemical, flag multicomponent chemicals, neutralize. All chemicals which are duplicates or flagged with errors (e.g., erroneous valences or charges) are then excluded from the result, but all erroneous or duplicate records are included into a protocol associated with a given dataset and available for review in the user interface. The proprietary AC software uses the P2Y_6_R agonist (EC_50_) data (244 molecules) at either a cutoff of 1 mM or 5 mM with multiple algorithms integrated into the web-based software to build classification models. The algorithms include: deep learning (DL), adaboost classifier (ada), Bernoulli NB (bnb), *k*-nearest neighbors (kNN) classifier, logistic regression (lreg), random forest classifier (rf), support vector classification (SVC), and XGBoost (XGB) classifier models with Extended Connectivity Fingerprint (ECFP)6 descriptors. In all cases, fivefold cross validation was performed except for deep learning for which we removed 20% of the training set, in a stratified manner for the classification models, and these were used as external test sets for models trained on the remainder of the data.

### Pharmacological assays

Hit compounds for pharmacological screening were purchased from MedChemExpress (MCE, Monmouth Junction, NJ, USA) and Millipore Sigma (St. Louis, MO, USA). Stock solutions (5 mM, DMSO) of the non-nucleotide test compounds were stored at − 20 °C. Selective P2Y_1_R agonist MRS2365 ([[(1*R*,2*R*,3*S*,4*R*,5*S*)-4-[6-amino-2-(methylthio)-9*H*-purin-9-yl]-2,3-dihydroxybicyclo-[3.1.0]hex-1-yl]methyl] diphosphoric acid mono ester trisodium salt) was from Tocris (Minneapolis, MN). UDP was from Millipore Sigma (St. Louis, MO, USA).

### Calcium mobilization assay

In order to identify potential agonists or antagonists for human P2Y_6_R or P2Y_1_R, hit compounds from our ML models were tested using the FLIPR assay with Calcium 6 dye kit (Molecular Devices, CA) in 1321N1 astrocytoma cells either with stably expressing hP2Y_6_R or hP2Y_1_R [22, 31]. Briefly, 1321N1-hP2Y_6_R or -hP2Y_1_R cells were grown in a 96-well black plate (2 × 10^4^cells/well) for 24 h. Cells were treated with different concentrations of antagonist in presence of calcium 6 dye for 45 min, and assays were performed with a FLIPR-Tetra System (Molecular Devices, CA). Ester-protected dye is absorbed into the cytoplasm during incubation, is cleaved, and binds to calcium. Intracellular calcium is released upon P2Y_6_R activation with UDP (100 nM final concentration), or P2Y_1_R activation with selective agonist MRS2365, and interacts with the dye which was monitored using a FLIPR-Tetra. For agonist screening, cells were incubated with dye for 45 min followed by addition of hit compounds which were diluted in 1 × Hanks balanced salt solution (HBSS) buffer with 20 mM HEPES buffer at fixed concentration (80 µM). The IC_50_ values for different antagonists or % of activation at 80 µM of agonist were determined using a three-parameter logistic equation in GraphPad Prism software (Version 10.1.1, GraphPad, San Diego, CA). The results are presented as mean ± SEM (*n* = 2–3), unless noted with each molecule [29].

### Competitive binding assay

Hit compounds identified using our ML models were tested in CHO-hP2Y_14_R cells [37] by fluorescent-based competitive binding assay. CHO cells stably expressing human P2Y_14_R were grown in 96-well plate and when cells were 80% confluent incubated with different hit compounds with a single concentration (400 µM) for 30 min at 37 °C in 5% CO_2_ incubator. Fluorescent antagonist MRS 4174 (20 nM) [37] was added, and incubation continued for another 30 min. Cells were washed thrice with DPBS and detached with Cellstripper (Corning, Glendale, AZ, USA) followed by resuspension in DPBS. Acquire the mean fluorescent intensity (MFI) using flowcytometry (CytoFLEX, Beckman Coulter, Brea, CA, USA), and determine the percentage of inhibition. The mean autofluorescence of cells was measured in the absence of the fluorescent ligand. The mean fluorescence intensity in the presence of fluorescent ligand was corrected by subtracting the autofluorescence. Data analysis was performed with GraphPad Prism software (Version 10.1.1, GraphPad, San Diego, CA, USA) and presented as mean ± SEM (n = 2–3) [37].

## Results

### Selection of compounds

**Fig. 2:**
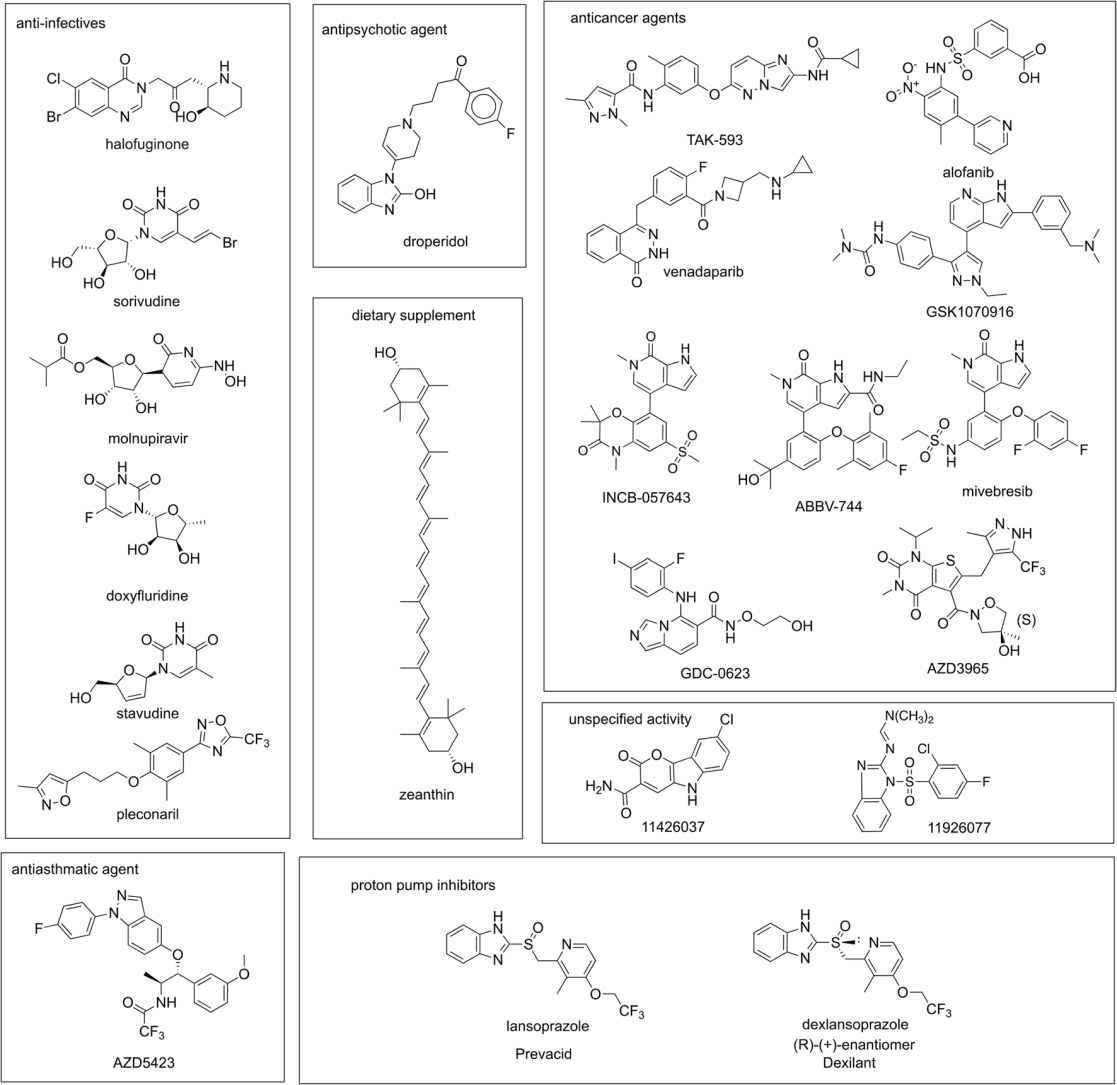
Structures of diverse compounds chosen for testing as ligands of the P2Y_6_R and other P2YRs. Compounds were selected using ML models with the exception of dexlansoprazole, mivebresib (ABBV-075), and INCB-057643, which were selected by similarity to other compounds tested

We used the public data available in the CHEMBL database for the P2Y_6_R agonists (EC_50_) (ChEMBL4714) to build classification machine learning models using the algorithms: DL, ada, bnb, kNN, lreg, rf, SVC, and XGB with our AC software at different cutoffs (Table 1). Models showed generally good fivefold cross validation statistics and we selected a model built with a cut-off at 5 μM to score the following compound libraries: Microsource (2560 compounds), CNS-Penetrant compound library (MCE, 833 compounds), clinical compound library (MCE, 1977 compounds), and compounds from our internal projects (> 200 molecules) using consensus predictions. We identified a small set of 19 candidate molecules for screening at P2Y_6_R (structures shown in Fig. 2). The structures include mostly known experimental and approved drugs, including anti-infective compounds, anticancer agents, an anti-asthmatic drug, an antipsychotic drug, proton pump inhibitors (PPIs), a dietary supplement, and several others available to us. Four of the antiviral agents have a uracil nucleoside-related structure. Three additional molecules were selected without ML.

**Table 1:** Machine learning classification model 5-fold cross validation statistics for P2Y_6_ agonists (EC_50_): (A) cut-off of 1 µM (83 actives, 161 inactives) and (B) cut-off of 5 µM (139 actives, 105 inactives)

Method	AUC	F1	Precision	Recall	Accuracy	Specificity	Cohen’s kappa	MCC
A								
DL	0.71	0.59	0.48	0.76	0.63	0.56	0.29	0.31
ada	0.69	0.36	0.54	0.29	0.67	0.87	0.17	0.19
bnb	0.77	0.59	0.61	0.58	0.73	0.81	0.40	0.40
kNN	0.77	0.61	0.62	0.61	0.75	0.81	0.42	0.43
lreg	0.77	0.57	0.64	0.53	0.73	0.84	0.38	0.39
rf	0.80	0.67	0.67	0.68	0.77	0.83	0.50	0.50
svc	0.79	0.63	0.59	0.68	0.73	0.76	0.42	0.42
xgb	0.81	0.65	0.68	0.63	0.77	0.84	0.48	0.48
B								
DL	0.72	0.73	0.69	0.79	0.67	0.52	0.32	0.32
ada	0.70	0.74	0.66	0.83	0.66	0.45	0.29	0.30
bnb	0.77	0.77	0.73	0.81	0.72	0.60	0.42	0.43
kNN	0.76	0.76	0.77	0.76	0.74	0.70	0.47	0.47
lreg	0.78	0.76	0.74	0.79	0.73	0.64	0.43	0.44
rf	0.79	0.76	0.82	0.72	0.75	0.79	0.50	0.51
svc	0.78	0.76	0.82	0.71	0.74	0.79	0.48	0.49
xgb	0.79	0.78	0.76	0.81	0.75	0.67	0.48	0.48

*DL* deep learning, *ada* adaboost classifier, *bnb* Bernoulli NB, *kNN* k-nearest neighbors classifier, *lreg* logistic regression, *rf* random forest classifier, *SVC* support vector classification, *XGB* XGBoost, *AUC* area under receiver operating characteristic curve, *F1* harmonic mean of precision and recall, *Precision* ratio of true positives to predicted positives, *Recall* ratio of true positives to all positives, *Accuracy* ratio of true predictions to all predictions, *Specificity* ratio of true negatives to all negatives, *Cohen’s kappa* a correlation coefficient which allows for the possibility that some correlation agreements happen by chance, *MCC* Matthew’s correlation coefficient which takes into account true and false positives and negatives to provide a correlation coefficient between observed and predicted binary classifications.

### Pharmacological evaluation

A total of 22 compounds (Fig. 2) were assembled for initial functional screening at the human (h) P2Y_6_R expressed in 1321N1 astrocytoma cells. Compounds were selected using ML models with the exception of dexlanzoprazole, mivebresib, and INCB-057643. The proton pump inhibitor (PPI) dexlansoprazole, which also has anti-fibrotic activity [38], was tested because its racemic form lansoprazole showed some activity initially. Mivebresib, a pan inhibitor of the bromodomain and extraterminal (BET) family of bromodomains [39], and INCB-057643 [40] were tested because they are both BET inhibitors and showed Tanimoto similarity (MACCS fingerprints) > 0.60 compared to anticancer drug ABBV-744 (0.61 for INCB-057643 and 0.65 for Mivebresib). The latter two compounds were selected after we discovered the P2Y-related activity of ABBV-744, a 6-methyl-7-oxo-6,7-dihydro-1*H*-pyrrolo[2,3-*c*]pyridine derivative that is a selective inhibitor of the BD2 domain of BET family [41]. Mivabresib and INCB-057643 have the same core heterocyclic structure as ABBV-744. In addition to anti-cancer activity, ABBV-744 also impedes SARS-CoV-2 infection by regulating the host response [42].

**Fig. 3:**
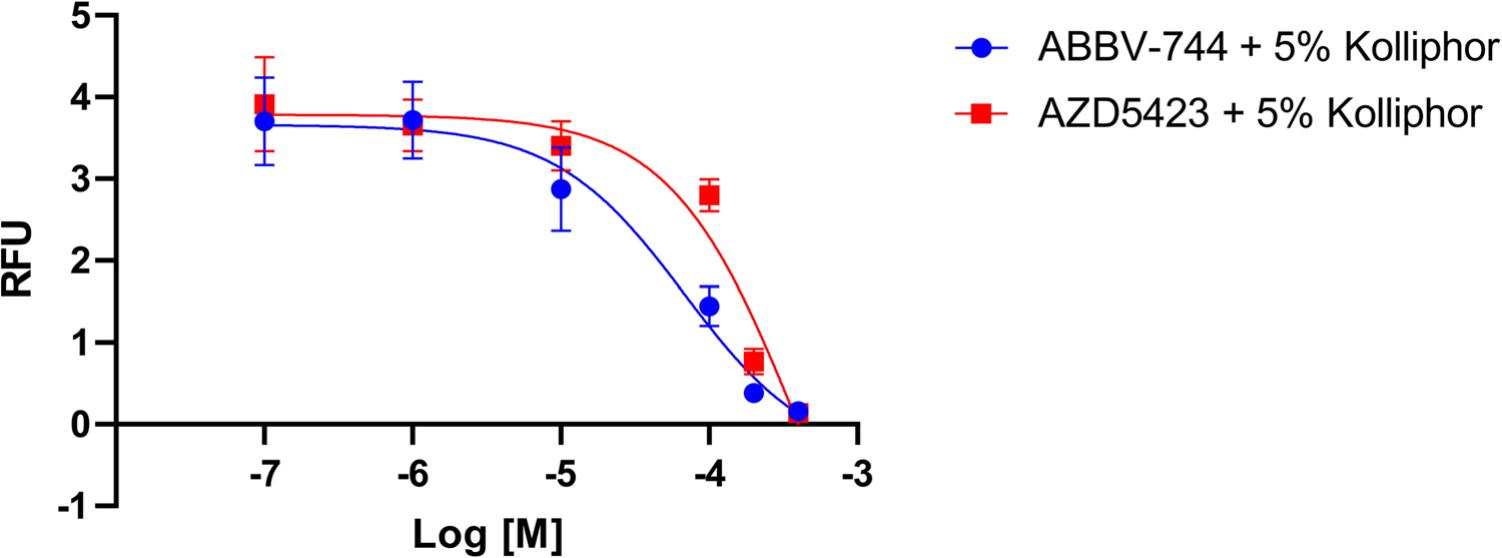
Concentration-dependent inhibition of the hP2Y_6_R, in the presence of a fixed concentration of agonist (UDP, 100 nM), by two hit compounds, ABBV-744 and AZD5423, measured using a FLIPR to detect calcium transients in stably transfected hP2Y_6_R-expressing 1321N1 astrocytoma cells (representative curves shown). The IC_50_ values determined are listed in Table 1 and in the text

The assay consisted of measuring the ability of each compound to inhibit calcium transients in the cell induced by the native P2Y_6_R agonist, UDP (200 nM, Table 2). The initial screen was at a single concentration (400 µM), which was set relatively high to lower the bar for detecting positive hits. Compounds that inhibited by > 50% at that concentration were run in full concentration–response curves to obtain an IC_50_ value. Two compounds (hit rate ~ 20%) displayed the most potent inhibition, thus warranting the determination. ABBV-744 and AZD5423 (having a 1-phenyl-1*H*-indazole scaffold) were found to have IC_50_ values of 75.7 µM and 574 µM, respectively (Fig. 3). Thus, ABBV-744 was the most interesting among the tested compounds as a putative P2Y_6_R antagonist. This compound is an experimental myelofibrosis and cancer drug [43] (clinicaltrials.gov, NCT04454658, accessed July 21, 2023) that acts as an inhibitor of BET bromodomain proteins, specifically BD2 domain of BRD2, BRD3 and BRD4. AZD5423 is an inhalable non-steroidal glucocorticoid receptor modulator that is in clinical trials for mild allergic asthma and COPD [44] (clinicaltrials.gov, NCT01226316, accessed July 21, 2023). There is no apparent structural similarity between the two uncharged P2Y_6_R antagonistic hit compounds, ABBV-744 and AZD5423.

**Table 2:** Assay of selected ML compounds at the hP2Y_6_R and the hP2Y_14_R

Compound	P2Y_6_R, mean IC_50_ ± SEM (µM) or % inhibition at 400 µM^a^	P2Y_6_R, % activation at 80 µM^b^	P2Y_14_, % inhibition at 100 µM (*n* = 3)
Putative P2Y_6_R antagonists (*n* = 3)			
ABBV-744^c^	75.7 ± 11.5	8.3 ± 1.4%	15 ± 1%
AZD5423^c^	574 ± 181	24 ± 6%	10 ± 3%
Inactive or weakly interacting with P2Y_6_R (*n* = 2)			
AZD3965^c^	12 ± 1%	4.9 ± 0.3%	30 ± 7%
Stavudine	66 ± 9%	22 ± 1%	1.3 ± 3.3%
Dexlansoprazole	31 ± 0%	11 ± 0%	6.0 ± 12%
Halofuginone	52 ± 0%	ND	0 ± 3%
11426037	47%	19 ± 5%	ND
Lansoprazole	41 ± 13%	11 ± 1%	4.2 ± 6.5%
Alofanib	31 ± 4%	23 ± 9%	17 ± 2%
Sorivudine	20 ± 11%	23 ± 2%	8.2 ± 10.4%
Molnupiravir	19 ± 11%	15 ± 1%	0 ± 9%
Doxifluridine	8.1 ± 1.4%	23 ± 1%	0 ± 13%
GDC-0623	14 ± 8%	24 ± 0%	0 ± 13%
Zeaxanthin	3.9%	26 ± 1%	0 ± 18%
GSK1070916	2.8 ± 2.8%	7.5 ± 1.9%	38 ± 2%
Venadaparib	0.1 ± 0.1%	13 ± 8%	15 ± 4%
Droperidol	3.6 ± 3.6%	10 ± 4%	19 ± 7%
Pleconaril	1.1 ± 1.1%	7.1 ± 0.0%	ND
TAK-593	8.1 ± 8.1%	7.1 ± 0.1%	50 ± 4%
11926077	0 ± 0%	15 ± 3%	ND
Mivebresib (ABBV-075)	17 ± 17%	14 ± 0%	13 ± 5%
INCB-057643	0%	0%	21 ± 2%

*ND* not determined.

^a^Inhibition of full activation of the P2Y_6_R by 100 nM UDP

^b^Activation by 100 nM UDP is considered 100%

^c^DMSO stock solution of the compound contained 5% Kolliphor HS-15 (Sigma, product 42966) by mass for solubility

The same compounds were tested in P2Y_6_R agonist mode, i.e., for the ability to stimulate calcium transients at 80 µM in the same stably transfected cell line in the absence of UDP. However, none of the compounds displayed significant, potential P2Y_6_R agonist activity. ABBV-744 and AZD5423 stimulated calcium transients to only 8.3% and 24%, respectively, relative to the full agonist (UDP, 200 nM) set as 100%. None of the compounds exceeded 26% increase of calcium transients at 80 µM. Therefore, ABBV-744 and AZD5423 are not partial agonists at the P2Y_6_R.

**Fig. 4:**
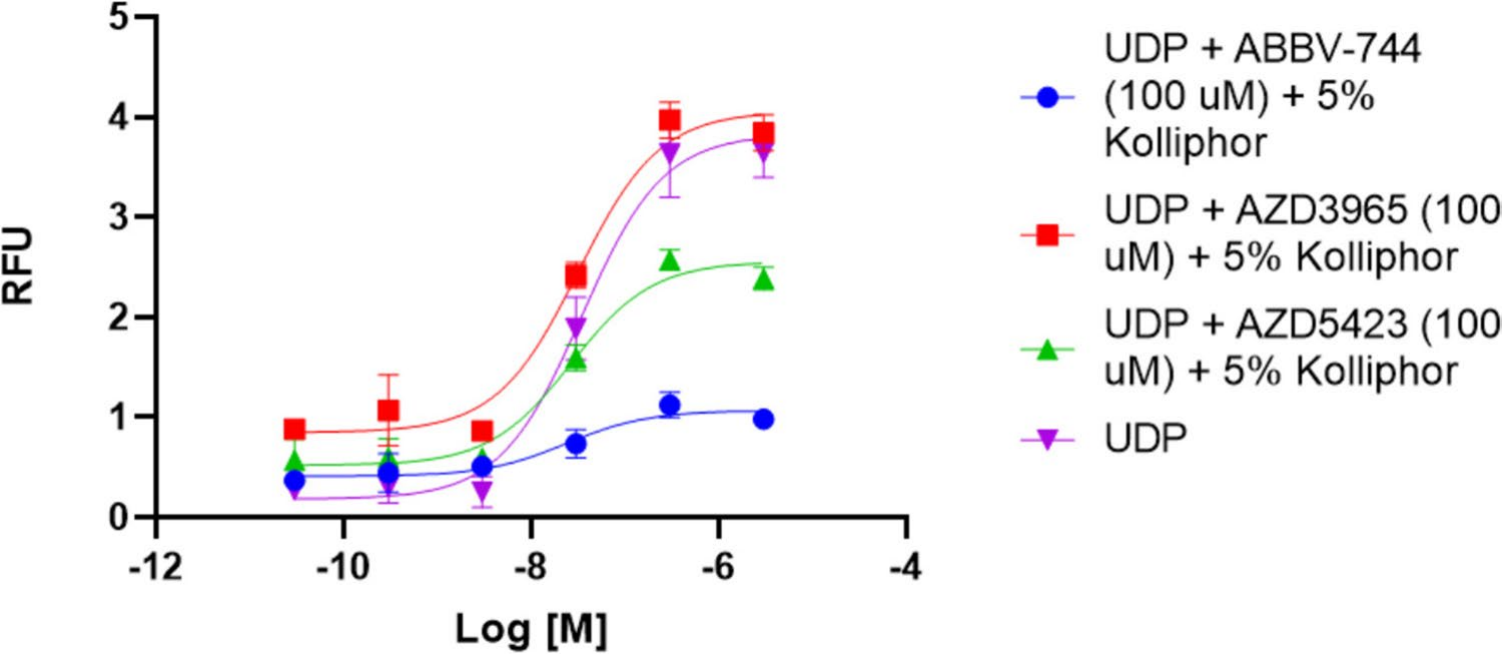
Concentration-dependent activation of the hP2Y_6_R by UDP and its inhibition by two hit compounds, ABBV-744 and AZD5423, measured using a FLIPR to detect calcium transients in stably transfected hP2Y_6_R-expressing 1321N1 astrocytoma cells (representative curves shown). AZD3965 failed to inhibit the UDP effect in this full curve experiment

The effects of increasing fixed concentrations of these two putative antagonists, ABBV-744 and AZD5423, on the concentration-dependent P2Y_6_R activation by UDP are shown in Fig. 4. The antagonism by both compounds appears to be insurmountable, suggesting that they are not acting as competitive P2Y_6_R antagonists.

In a previous study by Puhl et al. of ML-selected ligands of adenosine receptors (ARs) [45], we found unanticipated interactions with other AR subtypes than the originally targeted A_1_AR subtype. Thus, we considered that there might be some overlap of activity at other P2YRs, in a similar fashion. The compounds were therefore examined in a binding assay at the G_i_-coupled P2Y_14_R, which is similar to the P2Y_6_R in that both are activated by uracil nucleotides, including UDP, although P2Y_14_R is G_i_-coupled in the P2Y_12_R-like subfamily of P2YRs. The binding assay, which we developed and have used extensively to screen for P2Y_14_R antagonists, is based on the inhibition of binding of a selective, high affinity fluorescent ligand MRS4174, containing AlexaFluor488 [37]. This ligand is used in a whole cell assay (stably transfected hP2Y_14_R-expressing CHO cells) in which competitive binding was measured by flow cytometry. Although two compounds were not included in the P2Y_14_R binding assay, none of those tested potently inhibited P2Y_14_R binding at a concentration of 100 µM. Experimental ophthalmic drug TAK-593 and experimental Aurora B/C kinase inhibitor GSK1070916 [46, 47] registered only 50% and 38% inhibition, respectively, of P2Y_14_R fluorescent binding at this concentration, and all of the other compounds tested produced < 20% inhibition. The inhibition by ABBV-744 was only 15%. We chose not to elevate the primary screening concentration to 400 µM, because of the previous observation of interference in the fluorescent binding with various compounds at > 100 µM concentrations.

**Fig. 5:**
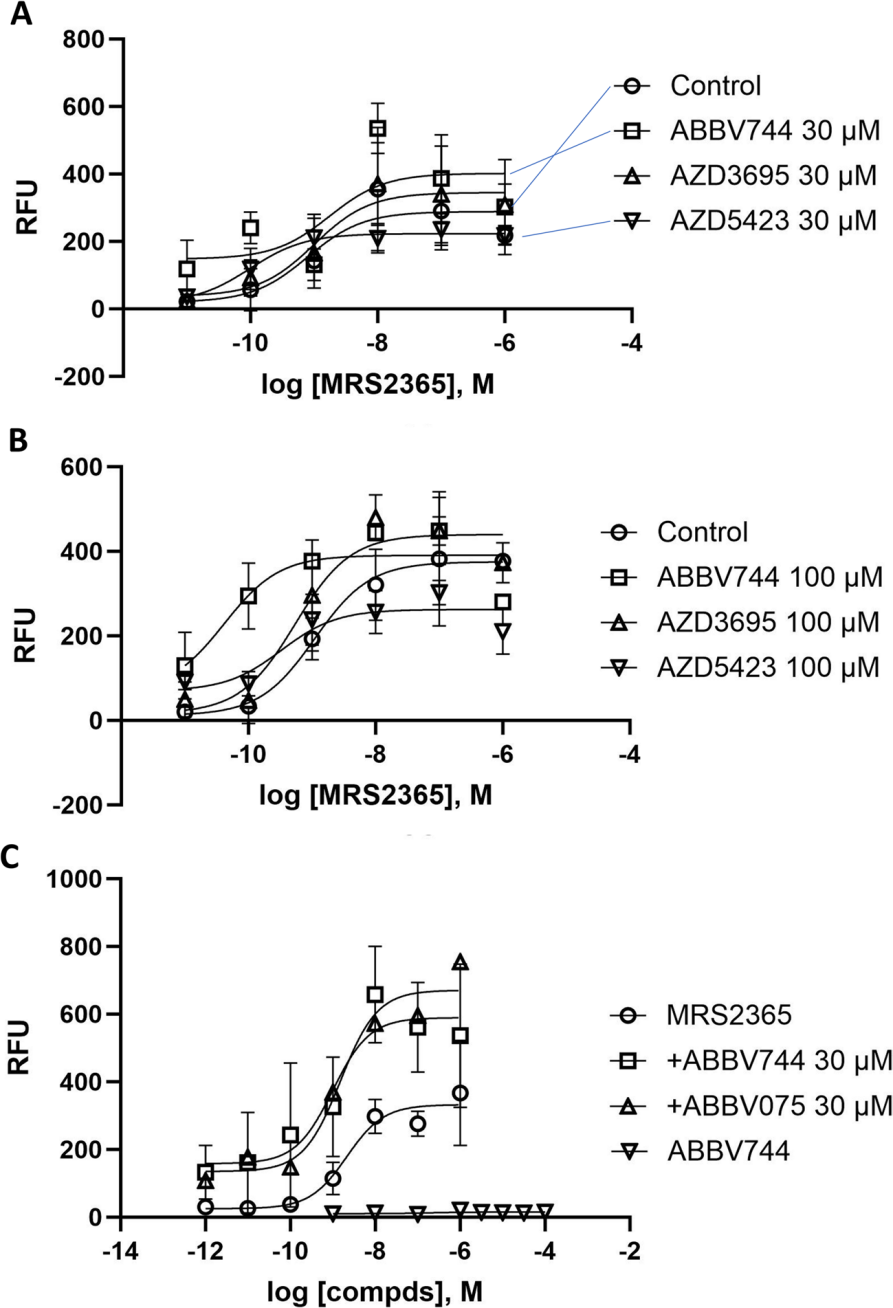
Comparison of functional effects (in a FLIPR assay of calcium transients) at the hP2Y_1_R stably expressed in 1321N1 astrocytoma cells. **A, B** Effects of ABBV-744 and several other ML-selected compounds on concentration-dependent hP2Y_1_R activation by selective nucleotide agonist MRS2365: **A** 30 µM ABBV-744, AZD3695, and AZD5423; **B** 100 µM ABBV-744, AZD3695, and AZD5423. **C** Effects of ABBV-744 and ABBV-075 on the activation of the hP2Y_1_R

Finally, the hit compounds were tested in a functional assay of P2Y_1_R activity (Fig. 5), as indicated by calcium transients in stably transfected hP2Y_1_R-expressing 1321N1 astrocytoma cells. Unexpectedly, ABBV-744 at 30 µM modestly enhanced activation of the P2Y_1_R by selective agonist MRS2365 (Fig. 5A). ABBV-744 at 100 µM produced a more robust enhancement of the P2Y_1_R activity (Fig. 5B). ABBV-075 at 30 µM appears to have a slight P2Y_1_R agonist activity (Fig. 5C).

## Conclusion

The principal hit compound, experimental anticancer drug ABBV-744, an epigenetic reader domain inhibitor, had multifaceted interactions with the P2YR family. It inhibits hP2Y_6_R activation by UDP in a non-surmountable fashion, suggesting that it is not a competitive antagonist based on Ca^2+^ mobilization, but additional studies of different signaling pathways will be needed. The precise mechanism of inhibition was not determined in this study and will be explored in later experiments. The same compound enhanced hP2Y_1_R activation by MRS2365, a selective agonist, but lacked orthosteric binding affinity at the hP2Y_14_R. Other ML-selected compounds were either weak (another anticancer drug, AZD5423) or inactive in inhibiting the hP2Y_6_R. We did not discover any novel hP2Y_6_R agonists, which was the initial ML strategy. Weakly inhibiting compounds at the hP2Y_14_R were TAK-593 and GSK1070916. Nevertheless, as in our previous study of ML for identifying adenosine receptors ligands [45], activity at closely related subtypes of the same GPCR family, or other atypical activities at the targeted subtype, seems to occur more often than not. Thus, we have identified new leads for using small molecules to modulate the P2Y_6_R as well as other P2YRs. The multifaceted activities of ABBV-744 need to be directly compared to other P2YRs not studied here, as well as other purinergic signaling proteins such as P2XRs. A ML approach such as that demonstrated has the potential to enable repurposing of approved or experimental drugs based on previously undetected activities.

## Data Availability

No datasets were generated or analyzed during the current study.

## Abbreviations

AC: Assay Central
DL: Deep learning
ada: Adaboost
bnb: Bernoulli NB
FLIPR: Fluorometric Imaging Plate Reader
HBSS: Hanks balanced salt solution
HEPES: 2-[4-(2-Hydroxyethyl)piperazin-1-yl]ethane-1-sulfonic acid
kNN: *k*-Nearest neighbors
lreg: Logistic regression
rf: Random forest
SVC: Support vector classification
XGB: XGBoost
GPCR: G protein-coupled receptor
PPI: Proton pump inhibitor
BET: Bromodomain and extraterminal domain

## Declarations

### Inclusion and diversity

We support inclusive, diverse, and equitable conduct of research.

## References

[1] Pándy-Szekeres G, Caroli J, Mamyrbekov A, Kermani AA, Keserű GM, Kooistra AJ, Gloriam DE (2023) GPCRdb in 2023: state-specific structure models using AlphaFold2 and new ligand resources. Nucleic Acids Res 51:D395–D402. 10.1093/nar/gkac1013 36395823 10.1093/nar/gkac1013 PMC9825476

[2] Congreve M, de Graaf C, Swain NA, Tate CG (2020) Impact of GPCR structures on drug discovery. Cell 181(1):81–91. 10.1016/j.cell.2020.03.003 32243800 10.1016/j.cell.2020.03.003

[3] Ballante F, Kooistra AJ, Kampen S, de Graaf C, Carlsson J (2021) Structure-based virtual screening for ligands of G protein–coupled receptors: what can molecular docking do for you? Pharmacol. Rev. 73(4):1698–1736. 10.1124/pharmrev.120.000246 10.1124/pharmrev.120.000246 34907092

[4] Sadybekov AV, Katritch V (2023) Computational approaches streamlining drug discovery. Nature 616:673–685. 10.1038/s41586-023-05905-z 37100941 10.1038/s41586-023-05905-z

[5] Nguyen ATN, Nguyen DTN, Koh HY, Toskov J, MacLean W, Xu A, Zhang D, Webb GI, May LT, Halls ML (2023) The application of artificial intelligence to accelerate G protein-coupled receptor drug discovery. Br. J. Pharmacol. 1– 14. 10.1111/bph.16140 10.1111/bph.16140 37161878

[6] Salmaso V, Jacobson KA (2020) Purinergic signaling: impact of GPCR structures on rational drug design. ChemMedChem 15:1958–1973. 10.1002/cmdc.202000465 32803849 10.1002/cmdc.202000465 PMC8276773

[7] Ekins S, Gerlach J, Zorn KM, Antonio BM, Lin Z, Gerlach A (2019a) Repurposing approved drugs as inhibitors of Kv7.1 and Nav1.8 to treat Pitt Hopkins Syndrome. Pharm. Res 36(9):137. 10.1007/s11095-019-2671-y 31332533 10.1007/s11095-019-2671-y PMC6814258

[8] Ekins S, Puhl AC, Zorn KM, Lane TR, Russo DP, Klein JJ, Hickey AJ, Clark AM (2019b) Exploiting machine learning for end-to-end drug discovery and development. Nat Mater 18(5):435–441. 10.1038/s41563-019-0338-z 31000803 10.1038/s41563-019-0338-z PMC6594828

[9] Ekins S, Mottin M, Ramos PRPS, Sousa BKP, Neves BJ, Foil DH et al (2020) Déjà vu: Stimulating open drug discovery for SARS-CoV-2. Drug Discov Today 25(5):928–941. 10.1016/j.drudis.2020.03.019 32320852 10.1016/j.drudis.2020.03.019 PMC7167229

[10] Mock M, Edavettal S, Langmead C, Russell A (2023) AI can help to speed up drug discovery - but only if we give it the right data. Nature 621(7979):467–470. 10.1038/d41586-023-02896-9 37726439 10.1038/d41586-023-02896-9

[11] Jacobson KA, Delicado EG, Gachet C, Kennedy C, von Kügelgen I, Li B, Miras-Portugal T, Novak I, Schöneberg T, Perez-Sen R, Thor D, Wu B, Yang Z, Müller CE (2020) Update of P2Y receptor pharmacology: IUPHAR Review: 27. Br J Pharmacol 177:2413–2433. 10.1111/bph.15005 32037507 10.1111/bph.15005 PMC7205808

[12] Koizumi S, Shigemoto-Mogam Y, Nasu-Tada K, Shinozaki Y, Ohsawa K, Tsuda M, Joshi BV, Jacobson KA, Kohsaka S, Inoue K (2007) UDP acting at P2Y_6_ receptors is a novel mediator of microglial phagocytosis. Nature 446:1091–1095 17410128 10.1038/nature05704 PMC3464483

[13] Lovászi M, Haas CB, Antonioli L, Pacher P, Haskó G (2021) The role of P2Y receptors in regulating immunity and metabolism. Biochem Pharmacol 187:114419. 10.1016/j.bcp.2021.114419 33460626 10.1016/j.bcp.2021.114419

[14] Umpierre AD, Li B, Ayasoufi K, Zhao S, Xie M, Thyen G, Hur B, Zheng J, Liang Y, Wu Z, Yu X, Sung J, Johnson AJ, Li Y, Wu LJ (2023) Microglial P2Y_6_calcium signaling promotes phagocytosis and shapes neuroimmune responses in epileptogenesis. bioRxiv 544691. 10.1101/2023.06.12.544691

[15] Oliveira-Giacomelli Á, Albino MC, de Souza HDN, Corrêa-Velloso J, de Jesus Santos AP, Baranova J, Ulrich H (2019) P2Y_6_ and P2X7 receptor antagonism exerts neuroprotective/neuroregenerative effects in an animal model of Parkinson’s disease. Front Cell Neurosci 13:476 31787881 10.3389/fncel.2019.00476 PMC6856016

[16] Milde S, van Tartwijk FW, Vilalta A et al (2021) Inflammatory neuronal loss in the substantia nigra induced by systemic lipopolysaccharide is prevented by knockout of the P2Y_6_receptor in mice. J Neuroinflammation 18:225. 10.1186/s12974-021-02280-2 34635136 10.1186/s12974-021-02280-2 PMC8504061

[17] Vieira RP, Müller T, Grimm M, von Gernler V, Vetter B, Dürk T, Cicko S, Ayata CK, Sorichter S, Robaye B, Zeiser R, Ferrari D, Kirschbaum A, Zissel G, Virchow JC, Boeynaems JM, Idzko M (2011) Purinergic receptor type 6 contributes to airway inflammation and remodeling in experimental allergic airway inflammation. Am J Respir Crit Care Med 184:215–223. 10.1164/rccm.201011-1762OC 21512170 10.1164/rccm.201011-1762OC

[18] Müller T, Fay S, Vieira RP, Karmouty-Quintana H, Cicko S, Ayata CK, Zissel G, Goldmann T, Lungarella G, Ferrari D, Di Virgilio F, Robaye B, Boeynaems JM, Lazarowski ER, Blackburn MR, Idzko M (2017) P2Y_6_ receptor activation promotes inflammation and tissue remodeling in pulmonary fibrosis. Front Immunol 8:1028. 10.3389/fimmu.2017.01028 28878780 10.3389/fimmu.2017.01028 PMC5572280

[19] Jain S, Pydi SP, Toti KS, Robaye B, Idzko M, Gavrilova O, Wess J, Jacobson KA (2020) Lack of adipocyte purinergic P2Y_6_ receptor greatly improves whole body glucose homeostasis. Proc Natl Acad Sci USA 117(48):30763–30774 33199639 10.1073/pnas.2006578117 PMC7720204

[20] Salem M, Lecka J, Pelletier J, Gomes Marconato D, Dumas A, Vallières L, Brochu G, Robaye B, Jobin C, Sévigny J (2022) NTPDase8 protects mice from intestinal inflammation by limiting P2Y_6_ receptor activation: identification of a new pathway of inflammation for the potential treatment of IBD. Gut 71:43–54. 10.1136/gutjnl-2020-320937 33452178 10.1136/gutjnl-2020-320937

[21] Zhou M, Wang W, Li Y, Zhang Q, Ji H, Li H, Hu Q (2020) The role of P2Y_6_R in cardiovascular diseases and recent development of P2Y_6_R antagonists. Drug Discovery Today 25:568–573. 10.1016/j.drudis.2019.12.015 31926135 10.1016/j.drudis.2019.12.015

[22] Maruoka H, Barrett MO, Ko H, Tosh DK, Melman A, Burianek LE, Balasubramanian R, Berk B, Costanzi S, Harden TK, Jacobson KA (2010) Pyrimidine ribonucleotides with enhanced selectivity as P2Y_6_ receptor agonists: Novel 4-alkyloxyimino, (S)-methanocarba, and 5′-triphosphate g-ester modifications. J Med Chem 53:4488–4501 20446735 10.1021/jm100287t PMC2935147

[23] Ginsburg-Shmuel T, Haas M, Schumann M, Reiser G, Kalid O, Stern N, Fischer B (2010) 5-OMe-UDP is a potent and selective P2Y_6_-receptor agonist. J Med Chem 53(4):1673–1685 20095577 10.1021/jm901450d

[24] Ginsburg-Shmuel T, Haas M, Grbic D, Arguin G, Nadel Y, Gendron FP, Reiser G, Fischer B (2012) UDP made a highly promising stable, potent, and selective P2Y_6_-Receptor agonist upon introduction of a boranophosphate moiety. Bioorg Med Chem 20:5483–5495. 10.1016/j.bmc.2012.07.042 22901672 10.1016/j.bmc.2012.07.042

[25] Toti KS, Jain S, Ciancetta A, Balasubramanian R, Charkaborty S, Surujdin R, Shi ZD, Jacobson KA (2017) Pyrimidine nucleotides containing a (S)-methanocarba ring as P2Y_6_ receptor agonists. Med Chem Commun 8:1897–1908 10.1039/c7md00397h PMC5798474 29423136

[26] Mamedova L, Joshi BV, Gao ZG, von Kügelgen I, Jacobson KA (2004) Diisothiocyanate derivatives as potent, insurmountable antagonists of P2Y_6_ nucleotide receptors. Biochem Pharmacol 67:1763–1770 15081875 10.1016/j.bcp.2004.01.011 PMC3413726

[27] Nishiyama K, Nishimura A, Shimoda K, Tanaka T, Kato Y, Shibata T, Tanaka H, Kurose H, Azuma YT, Ihara H, Kumagai Y, Akaike T, Eaton P, Uchida K, Nishida M (2022) Redox-dependent internalization of the purinergic P2Y_6_ receptor limits colitis progression. Sci. Signaling 15, eabj0644 10.1126/scisignal.abj0644 10.1126/scisignal.abj0644 35015570

[28] Ito M, Egashira S, Yoshida K, Mineno T, Kumagai K, Kojima H, Okabe T, Nagano T, Ui M, Matsuoka I (2017) Identification of novel selective P2Y_6_ receptor antagonists by high-throughput screening assay. Life Sci 180:137–142. 10.1016/j.lfs.2017.05.017 28527783 10.1016/j.lfs.2017.05.017

[29] Jung YH, Shah Q, Lewicki SA, Pramanik A, Gopinatth V, Pelletier J, Sévigny J, Iqbal J, Jacobson KA (2022) Synthesis and pharmacological characterization of multiply substituted 2H-chromene derivatives as P2Y_6_ receptor antagonists. Bioorg Med Chem Lett 75:128981. 10.1016/j.bmcl.2022.128981 36089113 10.1016/j.bmcl.2022.128981 PMC9555146

[30] Zhu Y, Zhou M, Cheng X et al (2023) Discovery of selective P2Y_6_R antagonists with high affinity and in vivo efficacy for inflammatory disease therapy. J Med Chem 66(9):6315–6332. 10.1021/acs.jmedchem.3c00210 37078976 10.1021/acs.jmedchem.3c00210

[31] Zhang D, Gao ZG, Zhang K, Kiselev E, Crane S, Wang J, Paoletta S, Yi C, Ma L, Zhang W, Han GW, Liu H, Cherezov V, Katritch V, Jiang H, Stevens RC, Jacobson KA, Zhao Q, Wu B (2015) Two disparate ligand-binding sites in the human P2Y_1_ receptor. Nature 520:317–321. 10.1038/nature14287 25822790 10.1038/nature14287 PMC4408927

[32] Li B, Han S, Wang M, Yu Y, Ma L, Chu X, Tan Q, Zhao Q, Wu B (2022) Structural insights into signal transduction of the purinergic receptors P2Y_1_R and P2Y_12_R. Protein Cell 14(5):382–386. 10.1093/procel/pwac025 10.1093/procel/pwac025 PMC10166156 37155313

[33] Rafehi M, Neumann A, Baqi Y, Malik EM, Wiese M, Namasivayam V, Müller CE (2017) Molecular recognition of agonists and antagonists by the nucleotide-activated G protein-coupled P2Y_2_ receptor. J Med Chem 60(20):8425–8440. 10.1021/acs.jmedchem.7b00854 28938069 10.1021/acs.jmedchem.7b00854

[34] Attah IY, Neumann A, Al-Hroub H, Rafehi M, Baqi Y, Namasivayam V (1864) Müller CE (2020) Ligand binding and activation of UTP-activated G-protein coupled P2Y2 and P2Y4 receptors elucidated by mutagenesis, pharmacological and computational studies. Biochim Biophys Acta (BBA) - Gen Subj 3:129501. 10.1016/j.bbagen.2019.129501 10.1016/j.bbagen.2019.129501 31812541

[35] Gaulton A, Bellis LJ, Bento AP, Chambers J, Davies M, Hersey A, Light Y, McGlinchey S, Michalovich D, Al-Lazikani B, Overington JP (2012) ChEMBL: a large-scale bioactivity database for drug discovery. Nucleic Acids Res 40:D1100–D1107. 10.1093/nar/gkr777 21948594 10.1093/nar/gkr777 PMC3245175

[36] Lane TR, Foil DH, Minerali E, Urbina F, Zorn KM, Ekins S (2021) Bioactivity comparison across multiple machine learning algorithms using over 5000 datasets for drug discovery. Mol Pharm 18:403–415. 10.1021/acs.molpharmaceut.0c01013 33325717 10.1021/acs.molpharmaceut.0c01013 PMC8237624

[37] Yu J, Ciancetta A, Dudas S, Duca S, Lottermoser J, Jacobson KA (2018) Structure-guided modification of heterocyclic antagonists of the P2Y_14_ receptor. J Med Chem 61:4860–4882. 10.1021/acs.jmedchem.8b00168 29767967 10.1021/acs.jmedchem.8b00168 PMC6428052

[38] Jiao Q, Zou F, Li S, Wang J, Xiao Y, Guan Z, Dong L, Tian J, Li S, Wang R, Zhang J, Li H (2022) Dexlansoprazole prevents pulmonary artery hypertension by inhibiting pulmonary artery smooth muscle cell to fibroblast transition. Am J Transl Res 14(8):5466–5479 36105026 PMC9452313

[39] Piha-Paul SA, Sachdev JC, Barve M, LoRusso P, Szmulewitz R, Patel SP, Lara PN Jr, Chen X, Hu B, Freise KJ, Modi D, Sood A, Hutti JE, Wolff J, O’Neil BH (2019) First-in-Human study of Mivebresib (ABBV-075), an oral pan-inhibitor of bromodomain and extra terminal proteins, in patients with relapsed/refractory solid tumors. Clin Cancer Res 25(21):6309–6319. 10.1158/1078-0432.CCR-19-0578 31420359 10.1158/1078-0432.CCR-19-0578

[40] Leal AS, Liu P, Krieger-Burke T, Ruggeri B, Liby KT (2020) The bromodomain inhibitor, INCB057643, targets both cancer cells and the tumor microenvironment in two preclinical models of pancreatic cancer. Cancers (Basel) 13(1):96. 10.3390/cancers13010096 33396954 10.3390/cancers13010096 PMC7794921

[41] Zhang L, Cai T, Lin X, Huang X, Bui MH, Plotnik JP, Bellin RJ, Faivre EJ, Kuruvilla VM, Lam LT, Lu X, Zha Z, Feng W, Hessler P, Uziel T, Zhang Q, Cavazos A, Han L, Ferguson DC, Mehta G, Shanmugavelandy SS, Magoc TJ, Rowe J, Goodwin NC, Dorritie KA, Boyiadzis M, Albert DH, McDaniel KF, Kati WM, Konopleva M, Shen Y (2021) Selective inhibition of the second bromodomain of BET family proteins results in robust antitumor activity in preclinical models of acute myeloid leukemia. Mol Cancer Ther 20(10):1809–1819. 10.1158/1535-7163.MCT-21-0029 34253595 10.1158/1535-7163.MCT-21-0029

[42] Samelson AJ, Tran QD, Robinot R, Carrau L, Rezelj VV, Kain AM, Chen M, Ramadoss GN, Guo X, Lim SA, Lui I, Nuñez JK, Rockwood SJ, Wang J, Liu N, Carlson-Stevermer J, Oki J, Maures T, Holden K, Weissman JS, Wells JA, Conklin BR, TenOever BR, Chakrabarti LA, Vignuzzi M, Tian R, Kampmann M (2022) BRD2 inhibition blocks SARS-CoV-2 infection by reducing transcription of the host cell receptor ACE2. Nat Cell Biol 24(1):24–34. 10.1038/s41556-021-00821-8 35027731 10.1038/s41556-021-00821-8 PMC8820466

[43] Faivre EJ, McDaniel KF, Albert DH, Mantena SR, Plotnik JP, Wilcox D, Zhang L, Bui MH, Sheppard GS, Wang L, Sehgal V, Lin X, Huang X, Lu X, Uziel T, Hessler P, Lam LT, Bellin RJ, Mehta G, Fidanze S, Pratt JK, Liu D, Hasvold LA, Sun C, Panchal SC, Nicolette JJ, Fossey SL, Park CH, Longenecker K, Bigelow L, Torrent M, Rosenberg SH, Kati WM, Shen Y (2020) Selective inhibition of the BD2 bromodomain of BET proteins in prostate cancer. Nature 578(7794):306–310. 10.1038/s41586-020-1930-8 31969702 10.1038/s41586-020-1930-8

[44] Werkström V, Prothon S, Ekholm E, Jorup C, Edsbäcker S (2016) Safety, pharmacokinetics and pharmacodynamics of the selective glucocorticoid receptor modulator AZD5423 after inhalation in healthy volunteers. Basic Clin Pharmacol Toxicol 119(6):574–581. 10.1111/bcpt.12621 27214145 10.1111/bcpt.12621

[45] Puhl AC, Gao ZG, Jacobson KA, Ekins S (2022) Machine learning for discovery of new ADORA modulators. Front Pharmacol 13:920643. 10.3389/fphar.2022.920643 35814244 10.3389/fphar.2022.920643 PMC9257522

[46] Mori Y, Yamamoto A, Nakagawa A, Hikima T, Isowaki A (2023) Potential of TAK-593 ophthalmic emulsion for the treatment of age-related macular degeneration. Biol Pharm Bull 46(7):921–928. 10.1248/bpb.b23-00066 37164692 10.1248/bpb.b23-00066

[47] Hardwicke MA, Oleykowski CA, Plant R, Wang J, Liao Q, Moss K, Newlander K, Adams JL, Dhanak D, Yang J, Lai Z, Sutton D, Patrick D (2009) GSK1070916, a potent Aurora B/C kinase inhibitor with broad antitumor activity in tissue culture cells and human tumor xenograft models. Mol Cancer Ther 8(7):1808–1817. 10.1158/1535-7163.MCT-09-0041 19567821 10.1158/1535-7163.MCT-09-0041

